# Fear of Birth Defects Is a Major Barrier to Soil-Transmitted Helminth Treatment (STH) for Pregnant Women in the Philippines

**DOI:** 10.1371/journal.pone.0085992

**Published:** 2014-02-26

**Authors:** Emily R. Insetta, Alex J. Soriano, Francis Isidore G. Totañes, Bernard J. C. Macatangay, Vicente Y. Belizario

**Affiliations:** 1 University of Pittsburgh School of Medicine, Pittsburgh, Pennsylvania, United States of America; 2 National Institutes of Health and College of Public Health, University of the Philippines Manila, Manila, Philippines; 3 Division of Infectious Diseases, University of Pittsburgh School Medicine, Pittsburgh, Pennsylvania, United States of America; Queensland Institute of Medical Research, Australia

## Abstract

The World Health Organization recommends anthelminthic treatment for pregnant women after the first trimester in soil-transmitted helminth (STH) endemic regions to prevent adverse maternal-fetal consequences. Although studies have shown the high prevalence of infection in the Philippines, no research has evaluated deworming practices. We hypothesized that pregnant women are not receiving deworming treatment and we aimed to identify barriers to World Health Organization guideline implementation. We conducted key informant interviews with local Department of Health (DOH) administrators, focus group discussions with nurses, midwives, and health care workers, and knowledge, attitudes, and practices surveys with women of reproductive age to elicit perspectives about deworming during pregnancy. Key informant interviews revealed that healthcare workers were not deworming pregnant women due to inadequate drug supply, infrastructure and personnel as well as fear of teratogenicity. Focus group discussions showed that healthcare workers similarly had not implemented guidelines due to infrastructure challenges and concerns for fetal malformations. The majority of local women believed that STH treatment causes side effects (74.8%) as well as maternal harm (67.3%) and fetal harm (77.9%). Women who were willing to take anthelminthics while pregnant had significantly greater knowledge as demonstrated by higher Treatment Scores (mean rank 146.92 versus 103.1, z = −4.40, p<0.001) and higher Birth Defect Scores (mean rank 128.09 versus 108.65, z = −2.43, p = 0.015). This study concludes that World Health Organization guidelines are not being implemented in the Philippines. Infrastructure, specific protocols, and education for providers and patients regarding anthelminthic treatment are necessary for the successful prevention of STH morbidity and mortality among pregnant women.

## Introduction

Soil-transmitted helminth (STH) infections, caused by *Ascaris*, *Trichuris*, and hookworms, are among the most prevalent neglected tropical diseases and are a leading cause of malnutrition in the developing world [1]. The World Health Organization (WHO) developed specific guidelines for pregnant women due to increased risk of comorbidities with STH infections. Helminth infection contributes to anemia in females of childbearing age and is associated with adverse maternal-fetal consequences including premature birth, low birth weight, and decreased breast milk production [2]. Current WHO guidelines for endemic regions indicate treatment for all adolescent females and pregnant women during the second and third trimester. A single dose of albendazole 400 mg or mebendazole 500 mg is recommended during the second trimester. In highly endemic regions, a second dose is recommended during the third trimester [3].

Many regions of the Philippines meet WHO criteria for endemicity for STH infections. A 2012 survey showed an overall prevalence of 30.4% (12.5%–61.8%) in adolescent females and 31.5% (13.2%–75.8%) in pregnant women. Of these infections, 7.9% (0.7%–22.6%) and 10.2% (0.9%–39.6%) were classified as heavy intensity infections, respectively [4]. Furthermore, a 2005 study showed that 55% of pregnant women in the Philippines were anemic [5].

The Philippines DOH published the Integrated Helminth Control Program (IHCP) Strategic Plan for 2006–2010. The plan outlined mass treatment strategies including biannual mass targeted deworming for all children younger than 12 years of age and annual selective deworming in endemic regions for special groups upon presentation to a health clinic. These high risk groups include adolescent females, pregnant women in the second or third trimester, soldiers, farmers, food handlers, and indigenous people [6], [7].

Despite the high prevalence of STH and anemia in Filipino women, there are no studies that assess the implementation of WHO guidelines for the pregnant population in the Philippines. Multiple studies in Uganda, Sierra Leone, and Peru confirmed that the use of anthelmintic medications during pregnancy decreased the prevalence of STH infections [8], [9], [10], [11]. A 2001 study in Sierra Leone showed that albendazole treatment during pregnancy successfully reduced the prevalence of maternal anemia [9].

Despite the efficacy of anthelmintic therapy during pregnancy, the Philippine DOH has faced challenges in implementing mass drug administration (MDA). The IHCP Strategic Plan described obstacles within healthcare agencies such as non-compliance, poor coverage of target populations, lack of sustainability and low prioritization of the STH program relative to other DOH initiatives such as vaccinations. Additional obstacles identified include poor access to clean water and sanitation as well as low acceptance among the targeted population [6]. The WHO indicated financial constraints, limited drug access, and lack of drug safety research as barriers to MDA in Western Pacific countries [5]. Low levels of awareness and knowledge among Filipinos were significantly associated with decreased acceptance of MDA programs [12]. Past initiatives have identified challenges to MDA, however, perspectives of providers and patients are needed to develop a successful deworming program for women of reproductive age.

The Philippines DOH is structured into national, regional, and local divisions designed to implement national health programs. The national STH coordinator oversees the distribution of medication, funding, and national guidelines. Regional STH coordinators work to provide communication between the local and national division in the DOH in order to improve effectiveness of health programs and respond to new challenges. The municipal health officers are local physicians hired by the DOH to implement national program at public health clinics termed Local Health Units (LHUs) [13].

In this study we surveyed healthcare providers and patients to determine current deworming practices and to identify potential barriers for treating pregnant women. We hypothesized that this population is not currently receiving anthelminthics and we aimed to identify major barriers to treatment.

## Methods

### Study Sites

**Figure 1:**
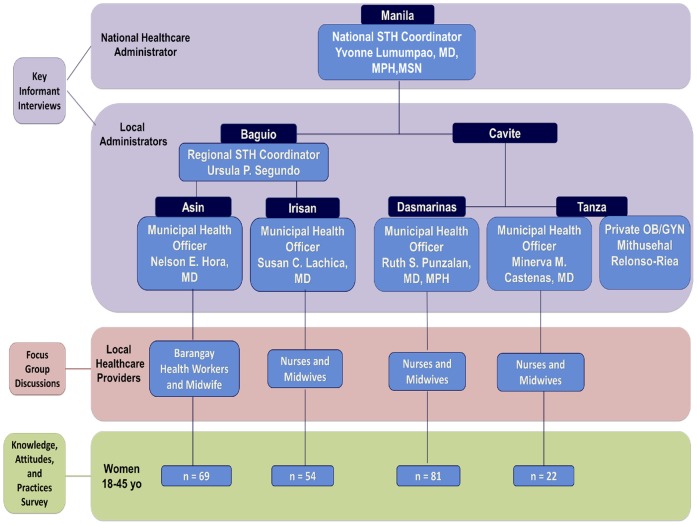
Study sites and participants. Key informant interviews were conducted with the National STH Coordinator, the Regional STH Coordinator and four local municipal health officers from Baguio City and Cavite. Focus group discussions with nurses, midwives, and barangay healthcare workers were conducted at each of the local health units along with Knowledge, Attitudes, and Practices surveys of women of reproductive age.

This study was conducted in June and July of 2011 in local health units (LHUs) of two regions of the Philippines: Baguio City and Cavite. The *barangays*, local government districts, in Baguio City included Asin and Irisan. In Cavite the selected municipalities were Dasmariñas and Tanza. An interview with the National STH Coordinator was conducted at the DOH in Manila, Philippines (Figure 1).

### Study Design

Key informant interviews (KIIs) were conducted with the DOH National STH coordinator, the Baguio regional STH coordinator, three local municipal health officers (MHOs) from Baguio, and four MHOs from Cavite. The KIIs elicited perspectives of government leaders regarding the prioritization and implementation of healthcare practices. In both Baguio and Cavite two Focus group discussions (FGDs) were conducted with nurses, midwives, and barangay health workers (BHWs) (Figure 1). The FGDs aimed to identify the local understanding of STH infection and treatment feasibility. Participants in the KIIs and FGDs were chosen based on their ongoing collaboration with the University of Philippines-Manila and NIH deworming initiative.

A cross-sectional Knowledge, Attitudes, and Practices (KAP) survey was administered to 226 voluntary participants ages 18 to 45 years old who presented for care at the local health clinics in Baguio City (n = 123) and Cavite (n = 103). Knowledge questions addressed women's understanding of modes of infection, treatment methods, and potential maternal or fetal harm from medications. Attitudes questions assessed willingness to take deworming medication while pregnant. Practice questions evaluated previous use of medications during pregnancy.

The English KAP survey was translated to Filipino and then back-translated to English to ensure consistency in cultural understanding of the survey tool. The survey was administered in either language based on participant preference. The KAP survey assessed local women's understanding of STH infections and their willingness to receive treatment.

### Ethical Considerations

This study received ethical approval from the University of Pittsburgh (PRO11030539) and the University of the Philippines – Manila (NIH 2011-023). Written informed consent was obtained from all study participants. The authors of this manuscript have given written informed consent (as outlined in the PLOS consent form) to publish these case details.

### Data Processing and Analysis

KII and FGD recordings were analyzed using the Grounded Theory approach [14]. This process involved analysis of audio files from two coders who independently elicited themes from the transcripts. The themes were compared and revised in order to ensure the reliability of the final results. For the KAP responses, frequency with percent distribution or mean with standard deviation was determined. The Mann-Whitney U test was used to correlate patients' knowledge of STH to their attitudes regarding treatment. A participant's knowledge level was determined by three scores including Infection Score, Treatment Score and Birth Defect Score with 32, 16, and 8 questions respectively. Scores were based on the sums of correct responses in each subject area. For the Infection Score, participants had to choose correct answers in response to questions of prevention, transmission, symptoms, and adverse effects with a maximum score of 32. For instance, if a woman identified abdominal pain as a symptom of infection, then she received one point. The Treatment Score was based on knowledge of therapies. For example, selecting mebendazole as an effective treatment was worth one point, and selecting papaya seeds as an ineffective treatment was also worth one point. A greater Birth Defect Score indicated that a participant thought deworming medications carried low risk of teratogenicity. For instance a woman received one point if she responded “no” to the question, “Will deworming medication cause physical deformity in your baby?”. The chi squared test (χ^2^) was used to correlate perceptions of side effects with attitudes regarding STH treatments. The comparison groups were divided into those with positive responses versus negative responses. Negative responses included “no,” “unsure,” or no response. All statistical analysis and data management were completed using SPSS Statistics 20.

## Results

### Key Informant Interviews

The national STH coordinator reported an abundant drug supply at the central DOH office, but described infrastructure challenges to drug delivery, especially in remote islands. She advocated for better communication between national and local leadership in order to distribute the available medications. The national coordinator was aware that the WHO guidelines to deworm pregnant women after the first trimester were not being implemented, so she thought it was feasible to aim for an initial goal of deworming 50% of the pregnant population. She stated the fear of teratogenicity among physicians was a greater barrier to STH treatment than fear among patients. She recommended prioritizing deworming pregnant women with positive stool samples and supplying drugs directly to LHUs.

The regional STH coordinator in Baguio City was also aware of WHO guidelines for deworming during pregnancy and cited patients' fear of teratogenicity as the primary barrier to deworming implementation. Due to patient concern and LHU non-compliance, the regional coordinator recommended incorporating deworming protocols into DOH guidelines for pregnant women and educating patients and providers (Table 1).

**Table 1:** STH Treatment for Pregnant Women: Perspectives of Health Care Providers.

	WHO Guideline Awareness and Implementation	Challenges	Recommendations
**National STH Coordinator**	Aware of WHO guidelines	Fear of teratogenicity among providers and patients	Prioritize deworming pregnant women with positive stool samples
	Excess drug supply	Poor infrastructure	Improve drug supply and communication for local health units
	50% coverage of pregnant women is reasonable target		
**Regional STH Coordinator**	Aware of WHO guidelines	Fear of teratogenicity among providers and patients	Include deworming guidelines in a DOH administrative order
	Lack of local compliance	Financial constraints in rural clinics	Publish an updated STH manual
			Expand the Maternal Newborn Child and Nutrition (MANCHAN) program to include deworming for pregnant women
			Educate and train health workers
**Municipal Health Officers**	Deworming pregnant women is not a priority	Fear of maternal-fetal consequences from medication	Provide evidence for deworming necessity and safety
	Detailed protocols and training are lacking	Insufficient drug safety evidence	Deworm at health clinics rather than home visits
		Pregnant women lack motivation to seek care	Provide a sustainable drug supply
		Non-cooperation of private physicians	Educate women
		Insufficient workforce	
		Poor drug supply	
**Nurses, Midwives, Barangay Health Workers**	Deworming medications are not given to pregnant women	Transportation and financial challenges of home visits	Train health workers
	Pregnant women with positive stool analyses are referred to physicians	Poor drug supply	Deworm at health clinics rather than home visits
	Deworming medications are teratogenic		Educate women

The National STH Coordinator, Regional STH Coordinator, and municipal health officers (MHOs) provided their perspectives during key informant interviews (KIIs). Nurses, midwives, and barangay health workers provided data during focus group discussions (FGDs).

The MHOs stated that insufficient evidence for albendazole/mebendazole safety during pregnancy and the lack of specific DOH protocols were the main barriers to STH treatment. In addition, they believed that the independent practice of non-government physicians, patients' lack of motivation to seek prenatal care, and challenges of home visits were barriers to MDA. Local providers felt that the DOH emphasized deworming and vaccination programs for children above STH treatment for pregnant women. They believed that low prioritization led to insufficient drug supply, workforce, and education.

To implement deworming programs, MHOs primarily requested drug safety evidence. One MHO said, “I attended a conference where deworming pregnant women was mentioned but not recommended because of concern for its side effects.” MHOs wanted education and training programs. Finally, they requested protocols for STH treatment in clinics rather than home visits, a sustainable drug supply, and education for their female patients.

### Focus Group Discussions

Nurses and midwives were either unaware or apprehensive regarding treatment of STH during pregnancy. In Cavite, a nurse said, “Deworming is not allowed for pregnant women,” and noted that she would refer a pregnant woman to a physician if she had a positive fecal analysis for STH infection. . In accordance with the views of MHOs, nurses and midwives in Baguio City said they were “anxious” about the uncertainty of teratogenic effects from deworming medications (Table 1).

Healthcare workers described many barriers to implementing WHO guidelines for STH treatment during pregnancy. Home visits have commonly been used for prenatal care and have been theorized as a potential vehicle for MDA. However, health care workers described home visits as “very challenging” due to difficult terrain, large coverage area, and financial constraints. Insufficient drug supply was also a primary concern. One health worker suggested that it would be “easier and better to give deworming medicines only to those with symptoms and positive stool exams.” Due to their apprehensions about drug safety and feasibility, healthcare workers requested “posters and advertisements” to educate local people about drug safety as well as “training about how to give deworming medicine” (Table 1).

### Knowledge, Attitudes, and Practices Surveys

A total of 226 women of reproductive age responded to the KAP survey with an average age of 29 years (Table 2). Forty-seven percent of the women were married and 25% were employed outside of the home. Forty-four percent of women completed high school and 28% had private or government health insurance. One-quarter of the women were pregnant and the average number of previous pregnancies was 2.39. The average household included 5 people and 2 rooms per home (Table 2).

**Table 2:** Characteristics of KAP survey participants.

Characteristics of Women of Reproductive Age	
Age, mean±SD	28.7±8.65
Married	46.9%
High School Education or Higher	44.2%
Employed	24.8%
Public or Private Health Insurance	27.9%
Currently Pregnant	25.2%
Previous Pregnancies, mean±SD	2.4±2.5
Household Size, mean±SD	4.8±2.4
Rooms in home, mean±SD	2.0±1.3

A total of 226 women of reproductive age completed a Knowledge, Attitudes, and Practices survey regarding STH infections during pregnancy. The majority of women surveyed were between the ages of 20–37 years old, unemployed, and did not complete a high school education.

The average score on all three knowledge scales was less than 50%. The mean Infection Score was 49.7% (15.9±5.64 out of 32), the mean Treatment Score was 13.4% (2.15±2.39 out of 16), and the mean Birth Defect Score was 7.3% (0.58±1.26 out of 8). Women who were willing to take deworming medication while pregnant had significantly higher Treatment Scores (mean rank 146.92 versus 103.1, z = −4.40, p<0.001) and Birth Defect Scores (mean rank 128.09 versus 108.65, z = −2.43, p = 0.015) than women unwilling to take STH medication during pregnancy. The Infection Score was not correlated with willingness to take deworming tablets while pregnant (Table 3).

**Table 3:** Soil-transmitted helminth (STH) knowledge score, by willingness to take deworming medication while pregnant.

Knowledge Score	Willing to take deworming medication while pregnant Mean Rank (n = 54)	Unwilling to take deworming medication while pregnant Mean Rank (n = 172)	z-value	p-value
**Modes of Infection**	121.15	111.10	−0.987	0.324
**Treatment Methods**	146.92	103.01	−4.40	**<0.001**
**Potential Birth Defects**	128.98	108.65	−2.43	**0.015**

The Mann-Whitney U Test was used to compare the mean knowledge scale scores to the “yes/no” responses to the question, “Would you be willing to take deworming medication while pregnant?” The 54 women who were willing to take deworming medications while pregnant had a greater knowledge of treatment methods and potential birth defects than the 172 women who were not willing to take deworming medications while pregnant.

Women of reproductive age who were willing to participate in a government deworming program had significantly higher Infection Scores (mean rank 120.58 versus 99.79, z = −2.27, p = 0.023) and Treatment Scores (mean rank 124.32 versus 92.56, z = −3.54, p<0.001) than those women who refused to be a part of the program (Table 4). Birth Defect Scores were not related to willingness to participate. In summary, knowledge of treatment and understanding of low teratogenic risk were associated with greater willingness to take deworming during pregnancy; meanwhile, knowledge of treatment and knowledge of infection were related to greater willingness to participate in a government deworming program.

**Table 4:** Soil-transmitted helminth (STH) knowledge scores, by willingness to participate in a government deworming program.

Knowledge Score	Willing to participate in a government sponsored deworming program Mean Rank (n = 149)	Unwilling to participate in a government sponsored deworming program Mean Rank (n = 77)	z-value	p-value
**Modes of Infection**	120.58	99.79	−2.27	**0.023**
**Treatment Methods**	124.32	92.56	−3.54	**<0.001**
**Potential Birth Defects**	118.05	104.70	−1.78	0.076

The Mann-Whitney U Test was used to compare the mean knowledge scale scores to the “yes/no” responses to the question, “Would you be willing to participate in a government-sponsored deworming program?” The 149 women who were willing to participate in a government sponsored deworming program had a higher knowledge of treatment methods and modes of infection than the 77 women who were not willing to participate in a government program.

The majority of women believed that STH treatment commonly causes side effects (74.8%) as well as maternal harm (67.3%) and fetal harm (77.9%). These perceptions were associated with an unwillingness to take anthelminthic medications during pregnancy (Table 5).

**Table 5:** Willingness to take deworming medication while pregnant, by perceived medication side effects.

		Side effects common from Medication (χ^2^ p = 0.003)		Harm to Fetus (χ^2^ p<0.001)		Harm to pregnant mother (χ^2^ p<0.001)	
		Yes	No	Yes	No	Yes	No
**Would you take deworming medications while pregnant?**	**Yes**	32 (14.2)	22 (9.7)	32 (14.2)	22 (9.7)	21 (9.3)	33 (14.6)
	**Negative**	137 (60.6)	35 (15.5)	144 (63.7)	28 (12.4)	131 (58.0)	41 (18.1)
	**Total**	169 (74.8)	57 (25.2)	176 (77.9)	50 (22.1)	152 (67.3)	74 (32.7)

The chi square test was used to compare the “yes/no” response to the question, “Would you take deworming while pregnant?” to their perception of side effect frequency, potential fetal harm, and potential maternal harm. A negative response includes no, unsure, and no response. Women who were not willing to take deworming medications while pregnant were more likely to believe that anthelminthics commonly cause adverse effects including maternal-fetal harm.

## Discussion

Providers' concerns were primarily founded in the lack of evidence for drug safety during pregnancy. These fears may be justified because although some evidence has shown deworming decreases infections and anemia in pregnant women, further human studies are needed to support the efficacy and safety of these medications. According to the Federal Drug Administration (FDA), albendazole and mebendazole are Category C drugs. Animal studies have shown embryotoxic effects and skeletal abnormalities in rats and rabbits [15]. A 1999 study of 7,087 Sri Lankan pregnant women showed that mebendazole did not increase the rate of birth defects [16]. In Israel, a 2003 prospective cohort study of 284 women determined that gestational mebendazole treatment did not increase major anomalies, miscarriages, stillbirths, or ectopic pregnancies [17]. Certain studies suggest that anthelmintics in pregnancy increase the incidence of eczema, but evidence remains unclear [8]. There is little research outside of these results, and no studies have been conducted in the Philippines. The WHO assumes that benefits of anthelmintics outweigh the risk of fetal anomalies specificallywhen administered in highly endemic regions after the first trimester.

Providers must first understand the safety of albendazole/mebendazole before they will advocate for the implementation of WHO guidelines. We recommend both disseminating evidence and increasing local research initiatives. Educational initiatives must target clinicians in endemic regions and emphasize correct timing for medication administration. An important component of developing an anthelminthic program for pregnant women will be to track outcomes including the presence or lack of adverse fetal effects.

In addition to uncertainty regarding drug safety, KIIs identified inadequate healthcare infrastructure as another primary barrier to STH treatment in pregnant women. FGDs with local health care workers suggested deworming pregnant women is not a DOH priority due to financial, workforce, and transportation constraints. The KAP surveys reinforced the qualitative findings from the KIIs and FGDs. The majority of local women believed STH treatment during pregnancy would cause harm to themselves and their babies.

A mass drug administration program relies on the knowledge and acceptance of the targeted population. Fear of teratogenicity among local women was primarily related to their lack of knowledge about STH infections, treatment, and drug safety. In a 2006 study in India, patient education significantly increased the knowledge of Lymphatic Filariasis (LF) as well as MDA coverage for LF in the targeted population [18]. Filipino women of reproductive age would benefit from a similar educational initiative.

It is important to note that there was inadequate knowledge even among the survey population that was limited to women who had access to medical care. Further studies could explore the perspectives of women in rural communities who may prefer traditional medicine or who may not seek health care.

As hypothesized, healthcare providers indicated that WHO guidelines were not being followed on a local level. Fear of teratogenicity among patients and providers was a major barrier. Therefore, education must target providers as well. The KIIs and FGDs were limited to providers at clinics with ongoing participation in a DOH deworming program. Health care workers from clinics without familiarity with STH treatment would likely have different perspectives.

In addition to fear of teratogenicity, LHUs lacked specific protocols and drug supplies for the sustainable implementation of WHO guidelines. Therefore, we also recommend incorporating specific protocols for deworming pregnant women into DOH guidelines, providing specific training for health workers, maintaining drug supply, and monitoring program efficacy. Before protocols can be implemented, however, addressing the fear of teratogenicity among providers and patients is the imperative first step to success in achieving optimal health outcomes for mothers and infants in the Philippines.

## Supporting Information

Appendix S1 **Key Informant Interview Script.** The script was used during Key Informant Interviews with healthcare providers including physicians and Department of Health officials.
(DOCX)

Appendix S2 **Focus Group Discussion Script.** The script was used during Focus Group Discussions with healthcare providers including nurses and barangay healthcare workers.
(DOCX)

Appendix S3 **Knowledge, Attitudes, and Practices Survey.** The survey was administered to women of reproductive age in Cavite and Baguio City in the Philippines.
(DOCX)
