# Supplementary material for: Fear of Birth Defects Is a Major Barrier to Soil-Transmitted Helminth Treatment (STH) for Pregnant Women in the Philippines
Source: PLoS One. 2014 Feb 26;9(2):e85992. doi: 10.1371/journal.pone.0085992 (PMC3935834; doi:10.1371/journal.pone.0085992)
Supplement: Appendix S1 — Key Informant Interview Script. The script was used during Key Informant Interviews with healthcare providers including physicians and Department of Health officials. (DOCX) [file pone.0085992.s001.docx]

**Appendix A. Key Informant Interview Script for Healthcare Officials**

1. What is your job title?_____________
2. How long have you worked in this position?______________
3. Age:_____
4. Sex: Male Female
5. What is your highest level of education?
   1. Elementary school
   2. High school
   3. College school
   4. Post-college
   5. Others (please specify): __________
6. What are the current guidelines to prevent soil-transmitted helminth infections for pregnant women?
7. What is the current standard of treatment for STH infections in pregnant women?
8. What are realistic goals for STH mass drug administration for pregnant women?
9. Are rural or urban populations of pregnant women is more at risk for STH infections? Why?
10. What is the most effective program plan to treat pregnant women most at risk?
11. In urban areas, who should administer the deworming medication to pregnant women?
12. In rural areas, who should administer the deworming medication to pregnant women?
13. In urban areas, how would the government monitor mass drug administration for pregnant women?
14. In rural areas, how would the government monitor mass drug administration for pregnant women?
15. In urban areas, what are the obstacles to implementing mass drug administration for pregnant women?
16. In rural areas, what are the obstacles to implementing mass drug administration for pregnant women?
