# Supplementary material for: Fear of Birth Defects Is a Major Barrier to Soil-Transmitted Helminth Treatment (STH) for Pregnant Women in the Philippines
Source: PLoS One. 2014 Feb 26;9(2):e85992. doi: 10.1371/journal.pone.0085992 (PMC3935834; doi:10.1371/journal.pone.0085992)
Supplement: Appendix S2 — Focus Group Discussion Script. The script was used during Focus Group Discussions with healthcare providers including nurses and barangay healthcare workers. (DOCX) [file pone.0085992.s002.docx]

**Appendix B. Focus Group Discussion Script for Healthcare Providers**

1. Have you ever provided deworming tablets to your patients?
2. What causes soil transmitted helminth infections?
3. What are the primary symptoms of soil transmitted helminth infections?
4. What are the best ways to treat soil transmitted helminth infections in pregnant women?
5. Are there any specific concerns for pregnant women and their babies with regard to soil transmitted helminth infections?
6. Do you think mass drug administration for pregnant women is a good idea? Why or why not?
7. Would you be willing to provide de-worming tablets to pregnant women? Why or why not?
8. If you wanted to provide de-worming tablets to pregnant women, what would make it hard for you to do so?
9. What are the biggest barriers to mass drug administration for pregnant women?
10. How might barriers to mass drug administration be different in rural versus urban areas?
11. What suggestions do you have for the government in establishing an effective mass drug administration program?
12. What would be the best location for distribution of de-worming medication for pregnant women?
13. What would be the best method to administer de-worming medication to pregnant women?
14. How do you think your pregnant patients feel about mass drug administration?
15. What might make it difficult for pregnant women to obtain de-worming medication?
