# Supplementary material for: Fear of Birth Defects Is a Major Barrier to Soil-Transmitted Helminth Treatment (STH) for Pregnant Women in the Philippines
Source: PLoS One. 2014 Feb 26;9(2):e85992. doi: 10.1371/journal.pone.0085992 (PMC3935834; doi:10.1371/journal.pone.0085992)
Supplement: Appendix S3 — Knowledge, Attitudes, and Practices Survey. The survey was administered to women of reproductive age in Cavite and Baguio City in the Philippines. (DOCX) [file pone.0085992.s003.docx]

**Appendix C. Knowledge, Attitudes, and Practices Survey for Women of Reproductive Age**

Please answer the following questions about yourself. Circle or write the correct answer.

1. Age: _______
2. Married: Yes No
3. The highest level of education that I have completed is:
4. Did not finish Elementary
5. Elementary
6. High School
7. Vocational school
8. College
9. None of the above
10. Others (please specify) _______
11. If my answer to question no. 2 is “Yes”, the highest level of education that my spouse has completed is:
    1. Did not finish Elementary
    2. Elementary
    3. High School
    4. Vocational school
    5. College
    6. None of the above
    7. Others (please specify) _______
12. Do you have a job? Yes No
13. If “Yes” what is your occupation?_____________________________
14. How many people live in your household (including yourself)?______
15. How many rooms are in your home?____________
16. Is there a toilet available at your home? Yes No Unsure
17. If “Yes” what type of toilet is present?
    1. No Toilet
    2. Pit Privy
    3. Water sealed toilet: Manual/Mechanical Flush
18. Do you have health insurance? Yes No Unsure
19. Are you currently pregnant? Yes No Unsure
20. Number of previous pregnancies: _______
21. If you were ever pregnant, what was your average number of prenatal visits per pregnancy to:

Doctors:_________

Nurses:__________

Midwives:________

Traditional Healers: ________

Others (please specify): ___________________

1. Would you be willing to visit a health center during your pregnancy? Yes No Unsure
2. With whom do you make prenatal healthcare decisions? (Encircle all that apply)
3. Spouse
4. Parents
5. Extended/other family members
6. Friends
7. Doctor/Healthcare Provider
8. Spiritual/Church Leader
9. None
10. Others (please specify): ________

For questions 17-22, please check the boxes that correspond to your answers.

1. What are the ways to adequately prevent common intestinal helminth infection?

|  | Yes | No | Unsure |
| --- | --- | --- | --- |
| Bathing everyday |  |  |  |
| Washing hands with soap and water |  |  |  |
| Washing hands with water only (no soap) |  |  |  |
| Keeping fingernails short |  |  |  |
| Washing food before eating |  |  |  |
| Wearing shoes |  |  |  |
| Others (please specify): __________ |  |  |  |

1. How can a person get infected by common intestinal helminth infection?

|  | Yes | No | Unsure |
| --- | --- | --- | --- |
| Poor personal hygiene |  |  |  |
| Mosquito bites |  |  |  |
| Use unclean water for cooking/drinking |  |  |  |
| Breathing polluted air |  |  |  |
| Waking barefoot |  |  |  |
| Others (please specify): __________ |  |  |  |

1. What are the symptoms of common intestinal helminth infection?

|  | Yes | No | Unsure |
| --- | --- | --- | --- |
| Abdominal pain |  |  |  |
| Diarrhea |  |  |  |
| Bruising |  |  |  |
| Loss of appetite |  |  |  |
| Loss of weight |  |  |  |
| Bloody stool |  |  |  |
| Fever |  |  |  |
| No Symptoms |  |  |  |
| Others (please specify): __________ |  |  |  |

1. What are the effects of intestinal helminth infection in pregnant women?

|  | Yes | No | Unsure |
| --- | --- | --- | --- |
| Weakness |  |  |  |
| Headaches |  |  |  |
| Miscarriage |  |  |  |
| Anemia |  |  |  |
| Decreased lactation |  |  |  |
| Death |  |  |  |
| Spiritual Consequences |  |  |  |
| Others (please specify): __________ |  |  |  |

1. What are the effects of intestinal helminth infection in newborn babies?

|  | Yes | No | Unsure |
| --- | --- | --- | --- |
| Premature Delivery |  |  |  |
| Low birth weight |  |  |  |
| Loss of appetite |  |  |  |
| Crying |  |  |  |
| Death |  |  |  |
| Spiritual Consequences |  |  |  |
| Others (please specify): __________ |  |  |  |

1. Would taking deworming medication harm your baby? Yes No Unsure

If “Yes”, in what ways would the medication harm your baby?

|  | Yes | No | Unsure |
| --- | --- | --- | --- |
| Early birth |  |  |  |
| Low birth weight |  |  |  |
| Physical Deformity |  |  |  |
| Mental Deformity |  |  |  |
| Death |  |  |  |
| Spiritual Consequences |  |  |  |
| Others (please specify): __________ |  |  |  |

For questions 23-54, please circle or write the correct answer.

1. Would you know if you have been infected with intestinal helminths/worms? Yes No Unsure
2. Have you ever been infected with intestinal helminths/worms? Yes No Unsure

If “Yes”, how many times?____

If “Yes”, how did you know?_________________

1. What are effective treatments for intestinal helminth infections?
   1. Ginger Yes No Unsure
   2. Papaya seeds Yes No Unsure
   3. Niyog-niyugan Yes No Unsure
   4. Prayer Yes No Unsure
   5. Mebendazole Yes No Unsure
   6. Albendazole Yes No Unsure
   7. Others (please specify): __________
2. Have you ever consulted the following health care providers for intestinal helminth infection?

| **Provider** | **Consulted for infection?** | **Did they help?** |
| --- | --- | --- |
| Doctor | Yes No Unsure | Yes No Unsure |
| Nurse | Yes No Unsure | Yes No Unsure |
| Midwife | Yes No Unsure | Yes No Unsure |
| Barangay Health Worker | Yes No Unsure | Yes No Unsure |
| Traditional Healers | Yes No Unsure | Yes No Unsure |
| Others (please specify): ____________________ |  | Yes No Unsure |

1. Have you ever been infected with helminth while pregnant? Yes No Unsure

If “Yes” how did you know?______________

If “Yes”, what did you do to treat the helminths?

- 1. Medication from Doctor/Hospital
  2. Medical Treatment from local witch doctors
  3. Prayer/Spiritual healing
  4. Family remedies
  5. Nothing
  6. Others (please specify): __________

1. Have you ever been offered deworming medicine? Yes No Unsure

If “Yes”, in what setting?

- 1. Clinic/Hospital
  2. School
  3. Home
  4. Others (please specify): __________

1. Have you ever refused deworming treatments? Yes No Unsure

If “Yes”, why did you refuse treatment? (Circle all that apply)

- 1. Risk of side effects
  2. Too expensive
  3. Felt healthy
  4. Others (please specify): __________

1. Are side effects from deworming medications common? Yes No Unsure
2. Would you take deworming medications while pregnant? Yes No Unsure
3. Would deworming tablets affect the health of your baby? Yes No Unsure
4. Would deworming medications make you sick? Yes No Unsure
5. Would you take deworming medications without testing/checkup for intestinal worms?

Yes No Unsure

1. Do you think your family will approve of you taking deworming drugs? Yes No Unsure
2. Would you take a deworming tablet if your family disapproved? Yes No Unsure
3. Would you take a deworming tablet if your husband disapproved? Yes No Unsure Not Married
4. Would you take deworming medication if you were feeling ill? Yes No Unsure
5. Would you take deworming medication if you were not feeling ill? Yes No Unsure
6. Would you take a deworming tablet if it were given to you by a doctor, nurse or midwife?

Yes No Unsure

1. Would you take a deworming tablet if it were given to you by a traditional healer?

Yes No Unsure

1. How much would you be willing to pay for deworming treatments?
   1. P 2.00
   2. P 5.00
   3. P 25.00
   4. P 45.00
   5. P 90.00
   6. None
2. Have you ever received information about intestinal worm/helminth infections from these sources?
   1. Television Yes No Unsure
   2. Radio Yes No Unsure
   3. Newspaper/magazine Yes No Unsure
   4. Billboard Yes No Unsure
   5. Pamphlet Yes No Unsure
   6. School Yes No Unsure
   7. Church Yes No Unsure
   8. Health center Yes No Unsure
   9. Others (please specify): __________
3. In the future, if you become infected with intestinal worms/helminths, what will you do for treatment?

If not pregnant? _______________________________________________________________

If pregnant? __________________________________________________________________

1. If you were treated for helminths in the past, would you need to be treated again?

Yes No Unsure

If yes, how many times would you need to be treated again? _____

1. Would you be willing to participate in a government sponsored deworming program?

Yes No Unsure

If “No”, then why not?

- 1. No trust in government
  2. No trust in health system/hospital
  3. Fear of side effects of medication
  4. Others (please specify): __________

1. What would be the easiest way for you to receive deworming medications?
2. At my Home
3. At the Hospital or Health Clinic
4. At Church
5. At a Pharmacy
6. At a nearby School
7. Others (please specify): __________
8. If deworming medications were offered to you, what would make it difficult for you to obtain the medicine?
9. How long (minutes) does it take for you to travel to the nearest health center? ­____
10. How do you get to the health center:
    1. Walking
    2. Public Transportation/Private Vehicle
    3. Others (please specify): __________
11. Is it inconvenient for you to go to the doctor? Yes No Unsure
12. Have you ever been to a doctor? Yes No Unsure
13. Would you choose to visit a traditional provider rather than a doctor/nurse? Yes No Unsure

If yes, why? __________________

1. Do you think the following statements better describe traditional healthcare providers (herbalists/herbolaryo, bulo-bulo healers, spiritistas, or hilots), doctors/nurses and midwives/BHWs?

|  | Circle that which is described. |
| --- | --- |
| Easier for me to access | Traditional Doctor/nurse Midwife/BHW Unsure |
| Easier for me to get there | Traditional Doctor/nurse Midwife/BHW Unsure |
| Lower cost | Traditional Doctor/nurse Midwife/BHW Unsure |
| More appropriate for treatment of severe illness | Traditional Doctor/nurse Midwife/BHW Unsure |
| More knowledge about diseases | Traditional Doctor/nurse Midwife/BHW Unsure |
| More spiritual knowledge | Traditional Doctor/nurse Midwife/BHW Unsure |
| Better treatment | Traditional Doctor/nurse Midwife/BHW Unsure |
| They understand me better. | Traditional Doctor/nurse Midwife/BHW Unsure |
| I trust them more. | Traditional Doctor/nurse Midwife/BHW Unsure |
